# Could *Helicobacter pylori* cause an abnormal immune response? 

**Published:** 2017

**Authors:** Genel Sur, Lucia Sur, Anamaria Girbovan, Gabriel Samasca, Giulia Alexandru, Rahela Carpa, Iulia Lupan

**Affiliations:** 1 *Department of Pediatrics II, Iuliu Hatieganu University of Medicine, Romania*; 2 *Department of Pediatrics I, Iuliu Hatieganu University of Medicine, Romania*; 3 *Department of Immunology II, Iuliu Hatieganu University of Medicine, Romania*; 4 *Emergency Hospital for Children, Romania*; 5 *Department of Microbiology, Babeş-Bolyai University, Romania*; 6 *Department of Molecular Biology and Biotechnology, Babeş-Bolyai University, Romania*

## To the Editor

 Rostami-Nejad et al and Samasca et al. found that the screening of *H. pylori* infection is necessary in patients with CD and iron deficiency (1,2). In a recent our study, the prevalence of *H. pylori* infection in our pediatric lot was 12%. 38 patients were positive for IgA *H. Pylori *antibodies*. *We performed IgA tissue transglutaminase antibodies to all 38 patients with positive results for IgA *H. pylori* antibodies, however, the results were negative. But, clinical diagnosis of children with *H. pylori* infection was diversified, moreover, nutritional diseases were the most frequent. *H. pylori* was involved in the following autoimmune diseases: type 1 diabetes mellitus (T1DM), thyroiditis and arthritis (Table 1). CD may be associated with *H. pylori* gastritis infection (3) however, few studies revealed this aspect. The association between *H. pylori* infection and thyroiditis in patients with T1DM was observed (4). *H. pylori* and patterns of gut microbiome have associated with low risk of T1DM (5). We also observed an association between *H. pylori* and T1DM in our study. Therefore, could autoimmunity be triggered by a decreased exposure to bacterial antigens? In our study, *H. Pylori *was associated with autoimmune diseases such as: type 1 diabetes mellitus, thyroiditis or arthritis but further studies are necessary for this etiology.

**Table 1:** Clinical diagnosis in patients with positive *H. pylori* infection

Diagnostic	Absolute frequency
Mild or moderate protein-energy malnutrition	5
Diabetes mellitus type 1	4
Gastritis/duodenitis	4
Nutritional anemia	3
Urticaria and erythema	2
Diarrhoea and gastroenteritis of presumed infectious origin	1
Bacterial infection	1
Benign tumor of the breast	1
Hereditary deficiency through missing factor VIII	1
Other coagulation abnormalities	1
Other bleeding disorders	1
Thyroiditis	1
Obesity	1
Pulmonary valve disorders	1
Other chronic obstructive pulmonary disease	1
Asthma	1
Acute appendicitis	1
Erythema nodosum	1
Other abnormalities in of pigmentation	1
Infectious arthropathy	1
Other arthritis	1
Back pain	1
Abnormal heart rhythm	1
Bleeding from respiratory system	1
Nausea and vomiting	1
TOTAL	38

## Conflict of interests

 The authors declare that they have no conflict of interest. 
